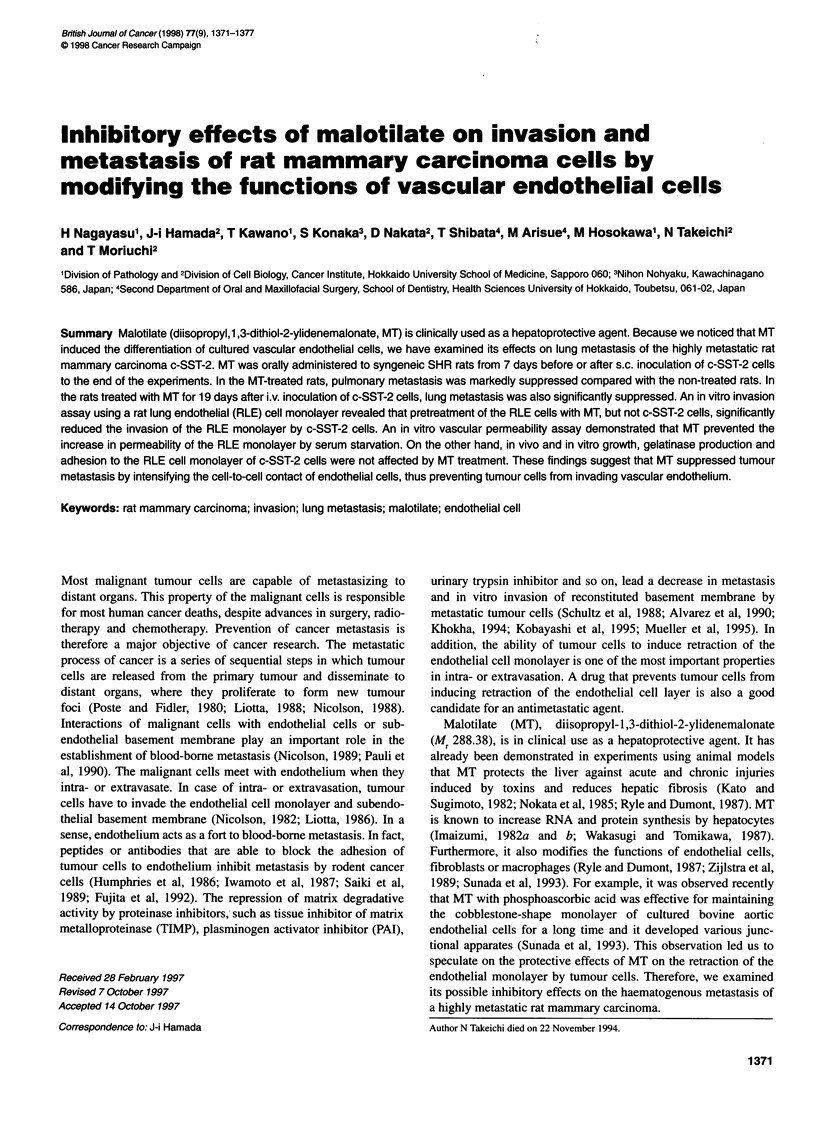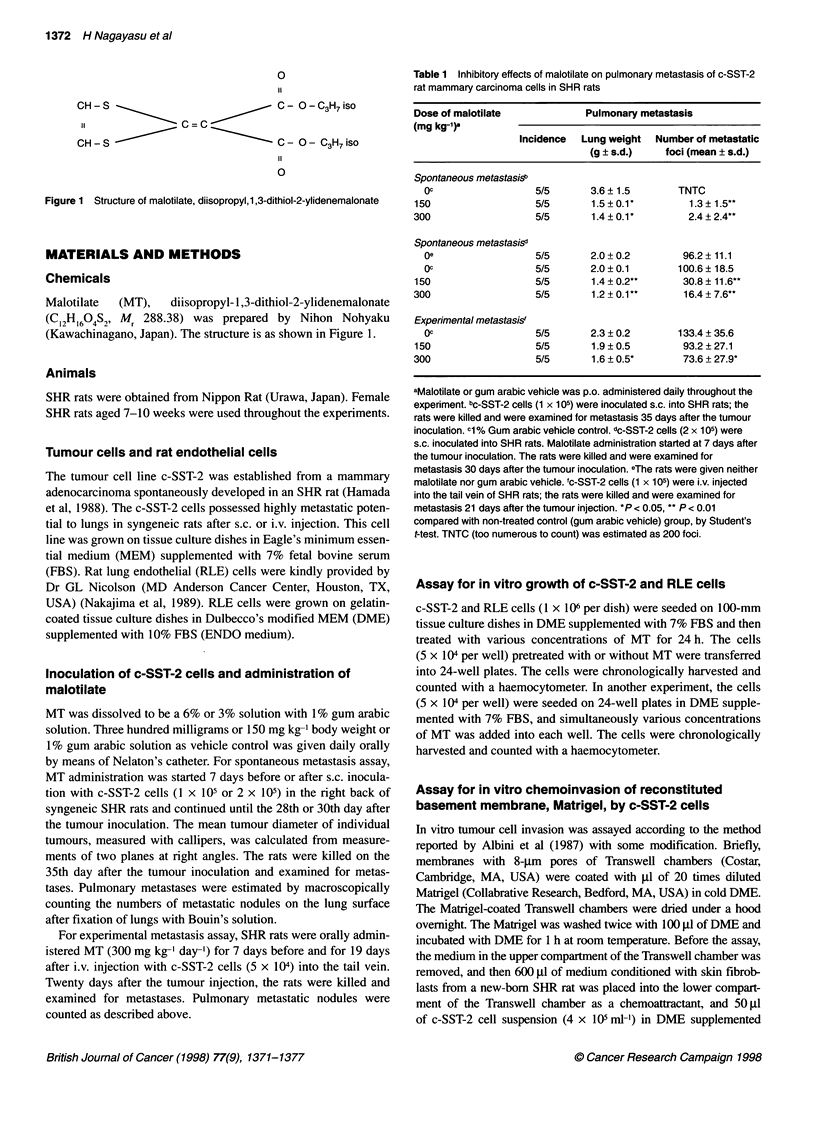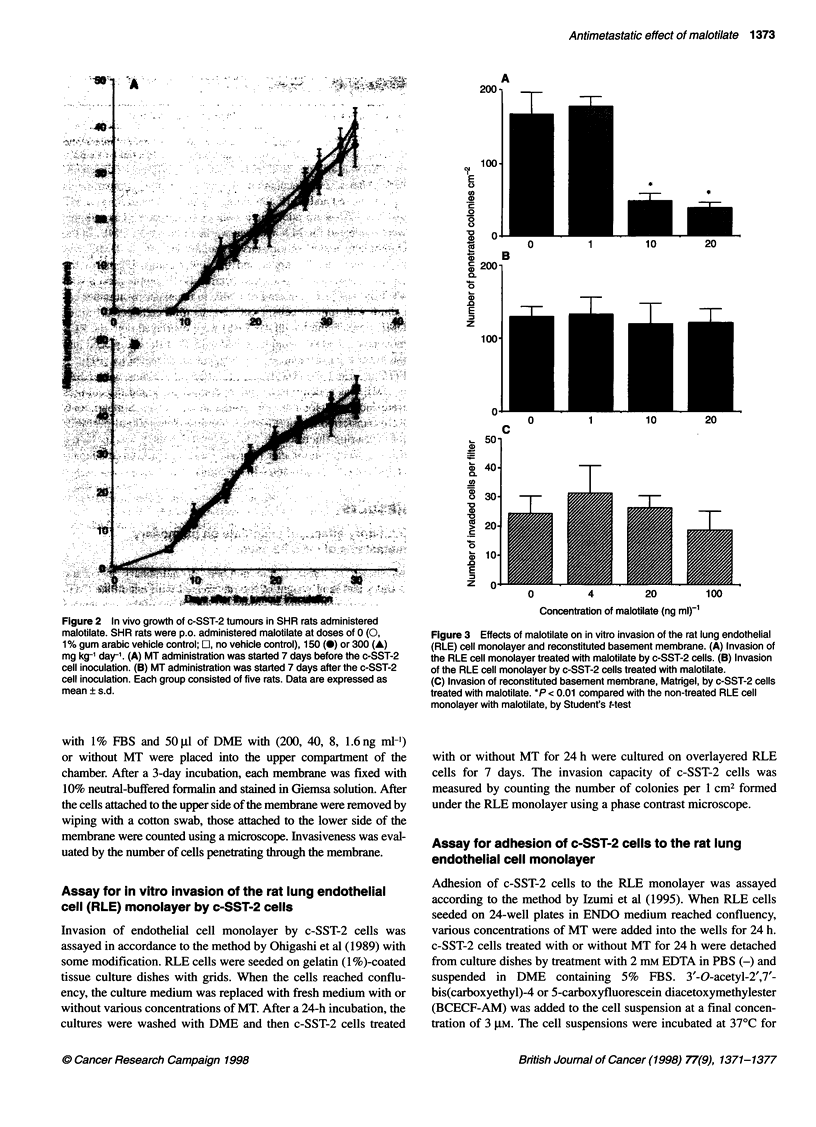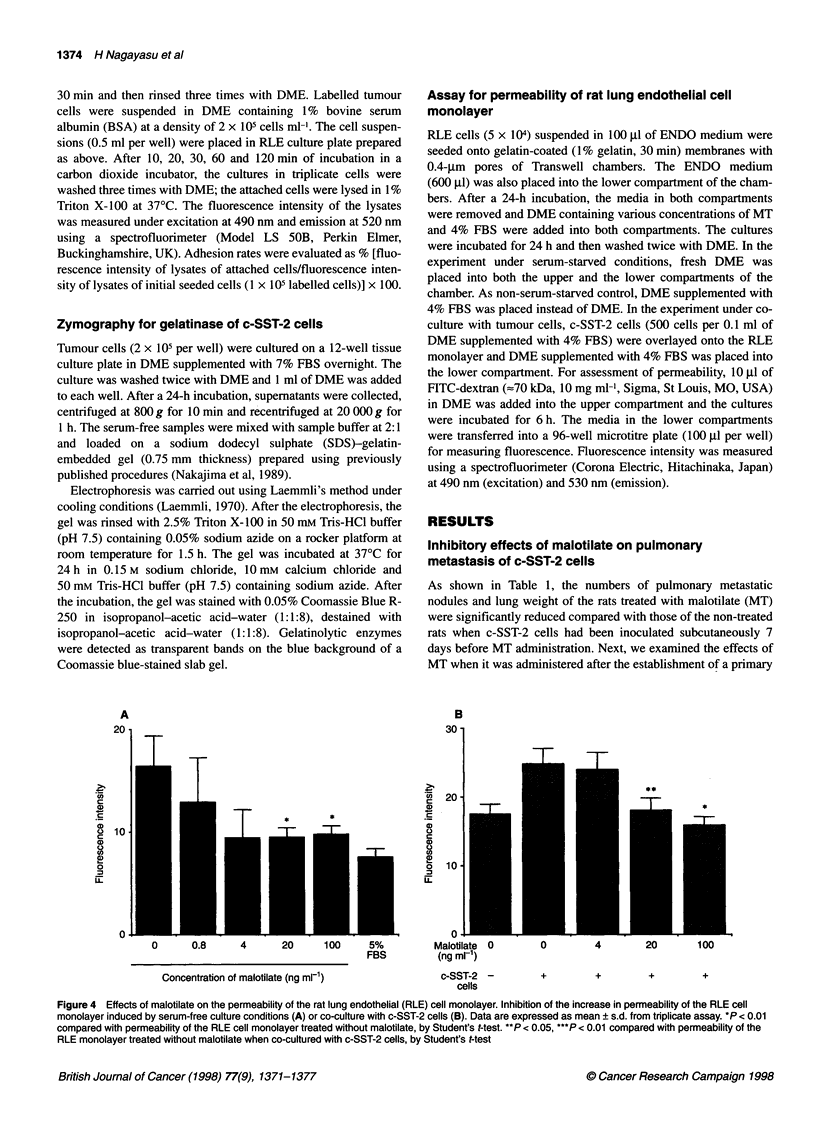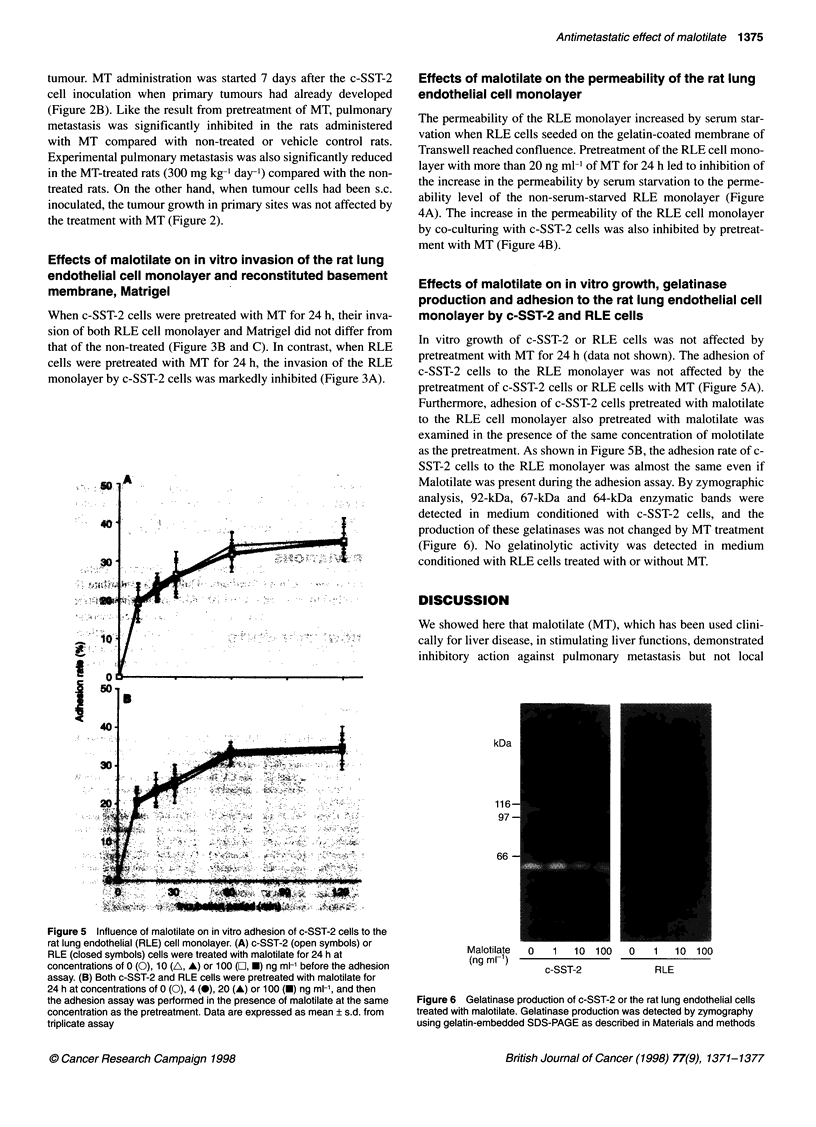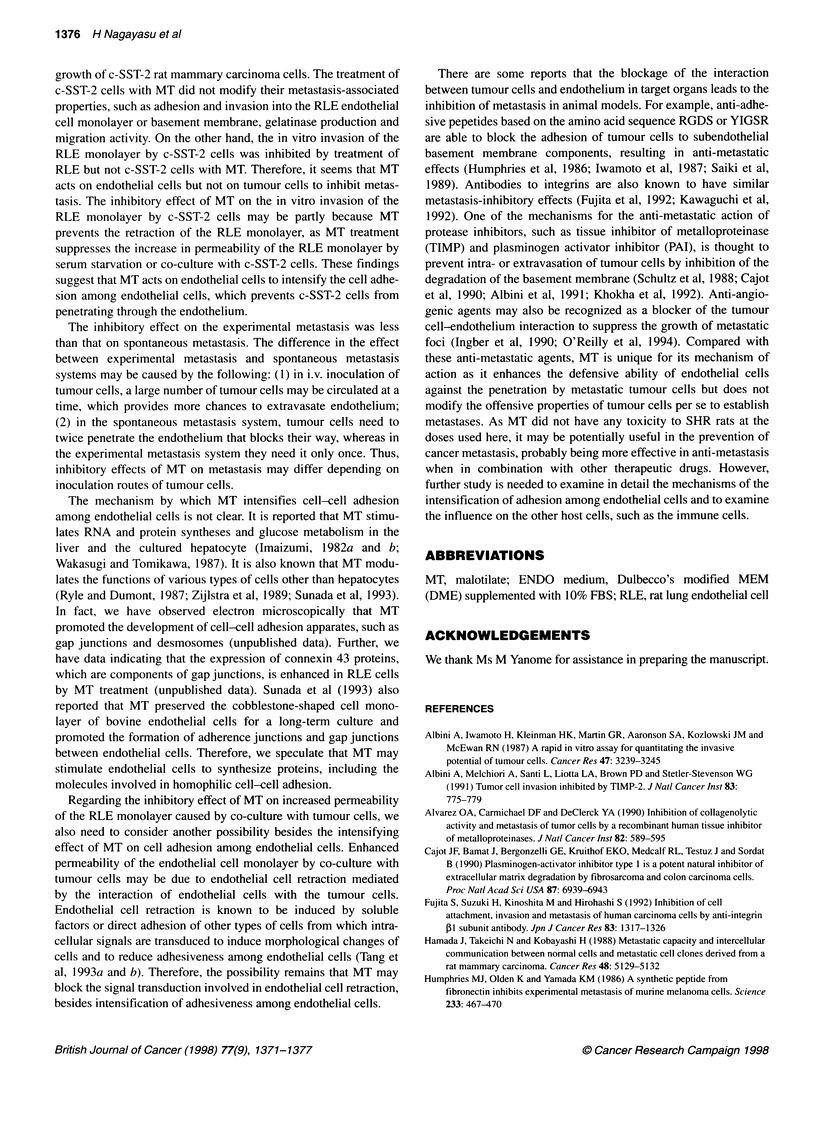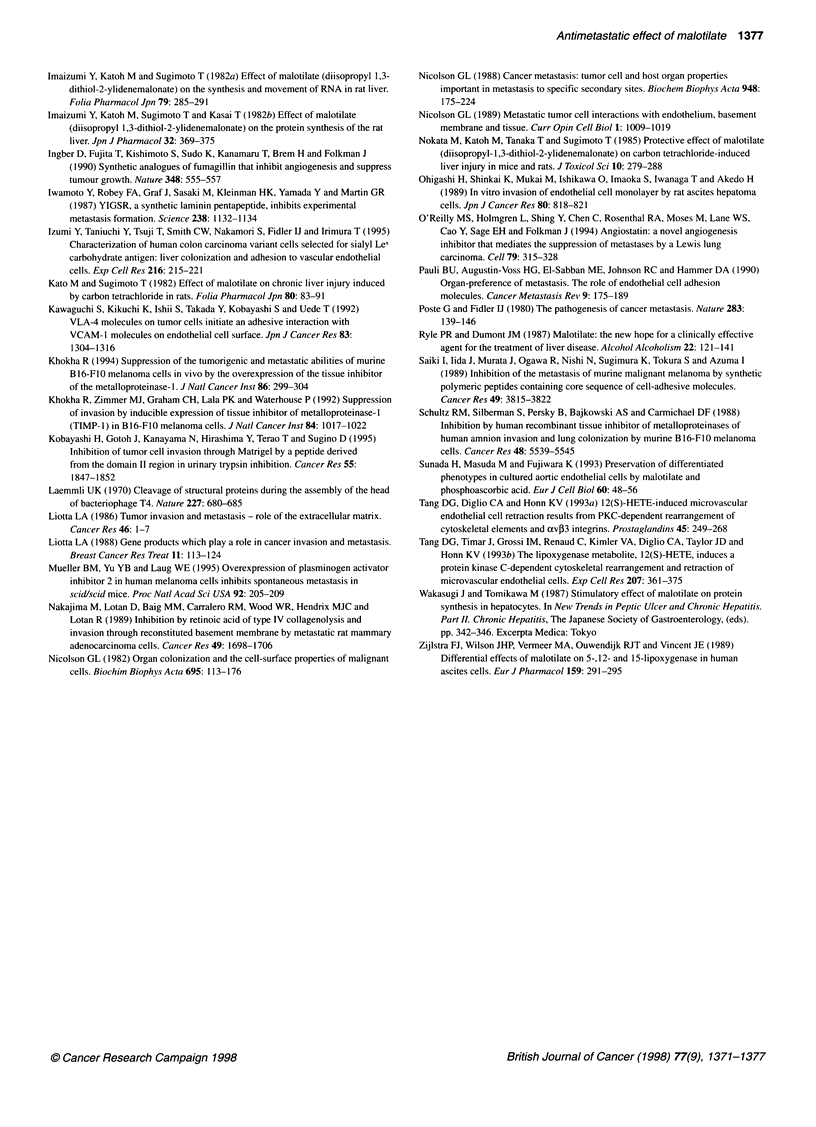# Inhibitory effects of malotilate on invasion and metastasis of rat mammary carcinoma cells by modifying the functions of vascular endothelial cells.

**DOI:** 10.1038/bjc.1998.229

**Published:** 1998-05

**Authors:** H. Nagayasu, J. Hamada, T. Kawano, S. Konaka, D. Nakata, T. Shibata, M. Arisue, M. Hosokawa, N. Takeichi, T. Moriuchi

**Affiliations:** Division of Pathology, Cancer Institute, Hokkaido University School of Medicine, Sapporo, Japan.

## Abstract

**Images:**


British Journal of Cancer (1998) 77(9), 1371–1377
© 1998 Cancer Research Campaign

### Inhibitory effects of malotilate on invasion and metastasis of rat mammary carcinoma cells by modifying the functions of vascular endothelial cells

**H Nagayasu[1], J-i Hamada[2], T Kawano[1], S Konaka[3], D Nakata[2], T Shibata[4], M Arisue[4], M Hosokawa[1], N Takeichi[2] and T Moriuchi[2]**

[1]Division of Pathology and [2]Division of Cell Biology, Cancer Institute, Hokkaido University School of Medicine, Sapporo 060; [3]Nihon Nohyaku, Kawachinagano 586, Japan; [4]Second Department of Oral and Maxillofacial Surgery, School of Dentistry, Health Sciences University of Hokkaido, Toubetsu, 061-02, Japan

**Summary** Malotilate (diisopropyl,1,3-dithiol-2-ylidenemalonate, MT) is clinically used as a hepatoprotective agent. Because we noticed that MT induced the differentiation of cultured vascular endothelial cells, we have examined its effects on lung metastasis of the highly metastatic rat mammary carcinoma c-SST-2. MT was orally administered to syngeneic SHR rats from 7 days before or after s.c. inoculation of c-SST-2 cells to the end of the experiments. In the MT-treated rats, pulmonary metastasis was markedly suppressed compared with the non-treated rats. In the rats treated with MT for 19 days after i.v. inoculation of c-SST-2 cells, lung metastasis was also significantly suppressed. An in vitro invasion assay using a rat lung endothelial (RLE) cell monolayer revealed that pretreatment of the RLE cells with MT, but not c-SST-2 cells, significantly reduced the invasion of the RLE monolayer by c-SST-2 cells. An in vitro vascular permeability assay demonstrated that MT prevented the increase in permeability of the RLE monolayer by serum starvation. On the other hand, in vivo and in vitro growth, gelatinase production and adhesion to the RLE cell monolayer of c-SST-2 cells were not affected by MT treatment. These findings suggest that MT suppressed tumour metastasis by intensifying the cell-to-cell contact of endothelial cells, thus preventing tumour cells from invading vascular endothelium.

**Keywords:** rat mammary carcinoma; invasion; lung metastasis; malotilate; endothelial cell

Most malignant tumour cells are capable of metastasizing to distant organs. This property of the malignant cells is responsible for most human cancer deaths, despite advances in surgery, radiotherapy and chemotherapy. Prevention of cancer metastasis is therefore a major objective of cancer research. The metastatic process of cancer is a series of sequential steps in which tumour cells are released from the primary tumour and disseminate to distant organs, where they proliferate to form new tumour foci (Poste and Fidler, 1980; Liotta, 1988; Nicolson, 1988). Interactions of malignant cells with endothelial cells or subendothelial basement membrane play an important role in the establishment of blood-borne metastasis (Nicolson, 1989; Pauli et al, 1990). The malignant cells meet with endothelium when they intra- or extravasate. In case of intra- or extravasation, tumour cells have to invade the endothelial cell monolayer and subendothelial basement membrane (Nicolson, 1982; Liotta, 1986). In a sense, endothelium acts as a fort to blood-borne metastasis. In fact, peptides or antibodies that are able to block the adhesion of tumour cells to endothelium inhibit metastasis by rodent cancer cells (Humphries et al, 1986; Iwamoto et al, 1987; Saiki et al, 1989; Fujita et al, 1992). The repression of matrix degradative activity by proteinase inhibitors, such as tissue inhibitor of matrix metalloproteinase (TIMP), plasminogen activator inhibitor (PAI), urinary trypsin inhibitor and so on, lead a decrease in metastasis and in vitro invasion of reconstituted basement membrane by metastatic tumour cells (Schultz et al, 1988; Alvarez et al, 1990; Khokha, 1994; Kobayashi et al, 1995; Mueller et al, 1995). In addition, the ability of tumour cells to induce retraction of the endothelial cell monolayer is one of the most important properties in intra- or extravasation. A drug that prevents tumour cells from inducing retraction of the endothelial cell layer is also a good candidate for an antimetastatic agent.

Malotilate (MT), diisopropyl-1,3-dithiol-2-ylidenemalonate ($M_r$ 288.38), is in clinical use as a hepatoprotective agent. It has already been demonstrated in experiments using animal models that MT protects the liver against acute and chronic injuries induced by toxins and reduces hepatic fibrosis (Kato and Sugimoto, 1982; Nokata et al, 1985; Ryle and Dumont, 1987). MT is known to increase RNA and protein synthesis by hepatocytes (Imaizumi, 1982*a* and *b*; Wakasugi and Tomikawa, 1987). Furthermore, it also modifies the functions of endothelial cells, fibroblasts or macrophages (Ryle and Dumont, 1987; Zijlstra et al, 1989; Sunada et al, 1993). For example, it was observed recently that MT with phosphoascorbic acid was effective for maintaining the cobblestone-shape monolayer of cultured bovine aortic endothelial cells for a long time and it developed various junctional apparates (Sunada et al, 1993). This observation led us to speculate on the protective effects of MT on the retraction of the endothelial monolayer by tumour cells. Therefore, we examined its possible inhibitory effects on the haematogenous metastasis of a highly metastatic rat mammary carcinoma.

*Received 28 February 1997*
*Revised 7 October 1997*
*Accepted 14 October 1997*

*Correspondence to:* J-i Hamada

Author N Takeichi died on 22 November 1994.

**Figure 1** Structure of malotilate, diisopropyl,1,3-dithiol-2-ylidenemalonate

**Table 1** Inhibitory effects of malotilate on pulmonary metastasis of c-SST-2 rat mammary carcinoma cells in SHR rats

| Dose of malotilate ($mg\ kg^{-1}$)[a] | Pulmonary metastasis | | |
|---|---|---|---|
| | Incidence | Lung weight (g ± s.d.) | Number of metastatic foci (mean ± s.d.) |
| *Spontaneous metastasis*[b] | | | |
| 0[c] | 5/5 | 3.6 ± 1.5 | TNTC |
| 150 | 5/5 | 1.5 ± 0.1* | 1.3 ± 1.5** |
| 300 | 5/5 | 1.4 ± 0.1* | 2.4 ± 2.4** |
| *Spontaneous metastasis*[d] | | | |
| 0[e] | 5/5 | 2.0 ± 0.2 | 96.2 ± 11.1 |
| 0[c] | 5/5 | 2.0 ± 0.1 | 100.6 ± 18.5 |
| 150 | 5/5 | 1.4 ± 0.2** | 30.8 ± 11.6** |
| 300 | 5/5 | 1.2 ± 0.1** | 16.4 ± 7.6** |
| *Experimental metastasis*[f] | | | |
| 0[c] | 5/5 | 2.3 ± 0.2 | 133.4 ± 35.6 |
| 150 | 5/5 | 1.9 ± 0.5 | 93.2 ± 27.1 |
| 300 | 5/5 | 1.6 ± 0.5* | 73.6 ± 27.9* |

[a]Malotilate or gum arabic vehicle was p.o. administered daily throughout the experiment. [b]c-SST-2 cells ($1 \times 10^5$) were inoculated s.c. into SHR rats; the rats were killed and were examined for metastasis 35 days after the tumour inoculation. [c]1% Gum arabic vehicle control. [d]c-SST-2 cells ($2 \times 10^5$) were s.c. inoculated into SHR rats. Malotilate administration started at 7 days after the tumour inoculation. The rats were killed and were examined for metastasis 30 days after the tumour inoculation. [e]The rats were given neither malotilate nor gum arabic vehicle. [f]c-SST-2 cells ($1 \times 10^5$) were i.v. injected into the tail vein of SHR rats; the rats were killed and were examined for metastasis 21 days after the tumour injection. *$P < 0.05$, ** $P < 0.01$ compared with non-treated control (gum arabic vehicle) group, by Student's *t*-test. TNTC (too numerous to count) was estimated as 200 foci.

### MATERIALS AND METHODS

### Chemicals

Malotilate (MT), diisopropyl-1,3-dithiol-2-ylidenemalonate ($C_{12}H_{16}O_4S_2$, $M_r$ 288.38) was prepared by Nihon Nohyaku (Kawachinagano, Japan). The structure is as shown in Figure 1.

### Animals

SHR rats were obtained from Nippon Rat (Urawa, Japan). Female SHR rats aged 7–10 weeks were used throughout the experiments.

### Tumour cells and rat endothelial cells

The tumour cell line c-SST-2 was established from a mammary adenocarcinoma spontaneously developed in an SHR rat (Hamada et al, 1988). The c-SST-2 cells possessed highly metastatic potential to lungs in syngeneic rats after s.c. or i.v. injection. This cell line was grown on tissue culture dishes in Eagle's minimum essential medium (MEM) supplemented with 7% fetal bovine serum (FBS). Rat lung endothelial (RLE) cells were kindly provided by Dr GL Nicolson (MD Anderson Cancer Center, Houston, TX, USA) (Nakajima et al, 1989). RLE cells were grown on gelatin-coated tissue culture dishes in Dulbecco's modified MEM (DME) supplemented with 10% FBS (ENDO medium).

### Inoculation of c-SST-2 cells and administration of malotilate

MT was dissolved to be a 6% or 3% solution with 1% gum arabic solution. Three hundred milligrams or 150 $mg\ kg^{-1}$ body weight or 1% gum arabic solution as vehicle control was given daily orally by means of Nelaton's catheter. For spontaneous metastasis assay, MT administration was started 7 days before or after s.c. inoculation with c-SST-2 cells ($1 \times 10^5$ or $2 \times 10^5$) in the right back of syngeneic SHR rats and continued until the 28th or 30th day after the tumour inoculation. The mean tumour diameter of individual tumours, measured with callipers, was calculated from measurements of two planes at right angles. The rats were killed on the 35th day after the tumour inoculation and examined for metastases. Pulmonary metastases were estimated by macroscopically counting the numbers of metastatic nodules on the lung surface after fixation of lungs with Bouin's solution.

For experimental metastasis assay, SHR rats were orally administered MT (300 $mg\ kg^{-1}\ day^{-1}$) for 7 days before and for 19 days after i.v. injection with c-SST-2 cells ($5 \times 10^4$) into the tail vein. Twenty days after the tumour injection, the rats were killed and examined for metastases. Pulmonary metastatic nodules were counted as described above.

### Assay for in vitro growth of c-SST-2 and RLE cells c-SST-2 and RLE cells ($1 \times 10^6$ per dish) were seeded on 100-mm tissue culture dishes in DME supplemented with 7% FBS and then treated with various concentrations of MT for 24 h. The cells ($5 \times 10^4$ per well) pretreated with or without MT were transferred into 24-well plates. The cells were chronologically harvested and counted with a haemocytometer. In another experiment, the cells ($5 \times 10^4$ per well) were seeded on 24-well plates in DME supplemented with 7% FBS, and simultaneously various concentrations of MT was added into each well. The cells were chronologically harvested and counted with a haemocytometer.

### Assay for in vitro chemoinvasion of reconstituted basement membrane, Matrigel, by c-SST-2 cells

In vitro tumour cell invasion was assayed according to the method reported by Albini et al (1987) with some modification. Briefly, membranes with 8-μm pores of Transwell chambers (Costar, Cambridge, MA, USA) were coated with μl of 20 times diluted Matrigel (Collabrative Research, Bedford, MA, USA) in cold DME. The Matrigel-coated Transwell chambers were dried under a hood overnight. The Matrigel was washed twice with 100 μl of DME and incubated with DME for 1 h at room temperature. Before the assay, the medium in the upper compartment of the Transwell chamber was removed, and then 600 μl of medium conditioned with skin fibroblasts from a new-born SHR rat was placed into the lower compartment of the Transwell chamber as a chemoattractant, and 50 μl of c-SST-2 cell suspension ($4 \times 10^5\ ml^{-1}$) in DME supplemented

 © Cancer Research Campaign 1998

**Figure 2** In vivo growth of c-SST-2 tumours in SHR rats administered malotilate. SHR rats were p.o. administered malotilate at doses of 0 (○, 1% gum arabic vehicle control; □, no vehicle control), 150 (●) or 300 (▲) mg kg$^{-1}$ day$^{-1}$. (**A**) MT administration was started 7 days before the c-SST-2 cell inoculation. (**B**) MT administration was started 7 days after the c-SST-2 cell inoculation. Each group consisted of five rats. Data are expressed as mean ± s.d.

**Figure 3** Effects of malotilate on in vitro invasion of the rat lung endothelial (RLE) cell monolayer and reconstituted basement membrane. (**A**) Invasion of the RLE cell monolayer treated with malotilate by c-SST-2 cells. (**B**) Invasion of the RLE cell monolayer by c-SST-2 cells treated with malotilate. (**C**) Invasion of reconstituted basement membrane, Matrigel, by c-SST-2 cells treated with malotilate. **P* < 0.01 compared with the non-treated RLE cell monolayer with malotilate, by Student's *t*-test with 1% FBS and 50 µl of DME with (200, 40, 8, 1.6 ng ml$^{-1}$) or without MT were placed into the upper compartment of the chamber. After a 3-day incubation, each membrane was fixed with 10% neutral-buffered formalin and stained in Giemsa solution. After the cells attached to the upper side of the membrane were removed by wiping with a cotton swab, those attached to the lower side of the membrane were counted using a microscope. Invasiveness was evaluated by the number of cells penetrating through the membrane.

### Assay for in vitro invasion of the rat lung endothelial cell (RLE) monolayer by c-SST-2 cells

Invasion of endothelial cell monolayer by c-SST-2 cells was assayed in accordance to the method by Ohigashi et al (1989) with some modification. RLE cells were seeded on gelatin (1%)-coated tissue culture dishes with grids. When the cells reached confluency, the culture medium was replaced with fresh medium with or without various concentrations of MT. After a 24-h incubation, the cultures were washed with DME and then c-SST-2 cells treated with or without MT for 24 h were cultured on overlayered RLE cells for 7 days. The invasion capacity of c-SST-2 cells was measured by counting the number of colonies per 1 cm$^2$ formed under the RLE monolayer using a phase contrast microscope.

### Assay for adhesion of c-SST-2 cells to the rat lung endothelial cell monolayer

Adhesion of c-SST-2 cells to the RLE monolayer was assayed according to the method by Izumi et al (1995). When RLE cells seeded on 24-well plates in ENDO medium reached confluency, various concentrations of MT were added into the wells for 24 h. c-SST-2 cells treated with or without MT for 24 h were detached from culture dishes by treatment with 2 mM EDTA in PBS (–) and suspended in DME containing 5% FBS. 3′-*O*-acetyl-2′,7′-bis(carboxyethyl)-4 or 5-carboxyfluorescein diacetoxymethylester (BCECF-AM) was added to the cell suspension at a final concentration of 3 µM. The cell suspensions were incubated at 37°C for

© Cancer Research Campaign 1998

30 min and then rinsed three times with DME. Labelled tumour cells were suspended in DME containing 1% bovine serum albumin (BSA) at a density of $2 \times 10^5$ cells $ml^{-1}$. The cell suspensions (0.5 ml per well) were placed in RLE culture plate prepared as above. After 10, 20, 30, 60 and 120 min of incubation in a carbon dioxide incubator, the cultures in triplicate cells were washed three times with DME; the attached cells were lysed in 1% Triton X-100 at 37°C. The fluorescence intensity of the lysates was measured under excitation at 490 nm and emission at 520 nm using a spectrofluorimeter (Model LS 50B, Perkin Elmer, Buckinghamshire, UK). Adhesion rates were evaluated as % [fluorescence intensity of lysates of attached cells/fluorescence intensity of lysates of initial seeded cells ($1 \times 10^5$ labelled cells)] × 100.

### Zymography for gelatinase of c-SST-2 cells

Tumour cells ($2 \times 10^5$ per well) were cultured on a 12-well tissue culture plate in DME supplemented with 7% FBS overnight. The culture was washed twice with DME and 1 ml of DME was added to each well. After a 24-h incubation, supernatants were collected, centrifuged at 800 ***g*** for 10 min and recentrifuged at 20 000 ***g*** for 1 h. The serum-free samples were mixed with sample buffer at 2:1 and loaded on a sodium dodecyl sulphate (SDS)–gelatin-embedded gel (0.75 mm thickness) prepared using previously published procedures (Nakajima et al, 1989).

Electrophoresis was carried out using Laemmli's method under cooling conditions (Laemmli, 1970). After the electrophoresis, the gel was rinsed with 2.5% Triton X-100 in 50 mM Tris-HCl buffer (pH 7.5) containing 0.05% sodium azide on a rocker platform at room temperature for 1.5 h. The gel was incubated at 37°C for 24 h in 0.15 M sodium chloride, 10 mM calcium chloride and 50 mM Tris-HCl buffer (pH 7.5) containing sodium azide. After the incubation, the gel was stained with 0.05% Coomassie Blue R-250 in isopropanol–acetic acid–water (1:1:8), destained with isopropanol–acetic acid–water (1:1:8). Gelatinolytic enzymes were detected as transparent bands on the blue background of a Coomassie blue-stained slab gel.

### Assay for permeability of rat lung endothelial cell monolayer

RLE cells ($5 \times 10^4$) suspended in 100 µl of ENDO medium were seeded onto gelatin-coated (1% gelatin, 30 min) membranes with 0.4-µm pores of Transwell chambers. The ENDO medium (600 µl) was also placed into the lower compartment of the chambers. After a 24-h incubation, the media in both compartments were removed and DME containing various concentrations of MT and 4% FBS were added into both compartments. The cultures were incubated for 24 h and then washed twice with DME. In the experiment under serum-starved conditions, fresh DME was placed into both the upper and the lower compartments of the chamber. As non-serum-starved control, DME supplemented with 4% FBS was placed instead of DME. In the experiment under co-culture with tumour cells, c-SST-2 cells (500 cells per 0.1 ml of DME supplemented with 4% FBS) were overlayed onto the RLE monolayer and DME supplemented with 4% FBS was placed into the lower compartment. For assessment of permeability, 10 µl of FITC-dextran (≈70 kDa, 10 mg $ml^{-1}$, Sigma, St Louis, MO, USA) in DME was added into the upper compartment and the cultures were incubated for 6 h. The media in the lower compartments were transferred into a 96-well microtitre plate (100 µl per well) for measuring fluorescence. Fluorescence intensity was measured using a spectrofluorimeter (Corona Electric, Hitachinaka, Japan) at 490 nm (excitation) and 530 nm (emission).

### RESULTS

### Inhibitory effects of malotilate on pulmonary metastasis of c-SST-2 cells

As shown in Table 1, the numbers of pulmonary metastatic nodules and lung weight of the rats treated with malotilate (MT) were significantly reduced compared with those of the non-treated rats when c-SST-2 cells had been inoculated subcutaneously 7 days before MT administration. Next, we examined the effects of MT when it was administered after the establishment of a primary

**Figure 4** Effects of malotilate on the permeability of the rat lung endothelial (RLE) cell monolayer. Inhibition of the increase in permeability of the RLE cell monolayer induced by serum-free culture conditions (**A**) or co-culture with c-SST-2 cells (**B**). Data are expressed as mean ± s.d. from triplicate assay. *$P < 0.01$ compared with permeability of the RLE cell monolayer treated without malotilate, by Student's *t*-test. **$P < 0.05$, ***$P < 0.01$ compared with permeability of the RLE monolayer treated without malotilate when co-cultured with c-SST-2 cells, by Student's *t*-test

© Cancer Research Campaign 1998

tumour. MT administration was started 7 days after the c-SST-2 cell inoculation when primary tumours had already developed (Figure 2B). Like the result from pretreatment of MT, pulmonary metastasis was significantly inhibited in the rats administered with MT compared with non-treated or vehicle control rats. Experimental pulmonary metastasis was also significantly reduced in the MT-treated rats (300 mg $kg^{-1}$ $day^{-1}$) compared with the non-treated rats. On the other hand, when tumour cells had been s.c. inoculated, the tumour growth in primary sites was not affected by the treatment with MT (Figure 2).

### Effects of malotilate on in vitro invasion of the rat lung endothelial cell monolayer and reconstituted basement membrane, Matrigel

When c-SST-2 cells were pretreated with MT for 24 h, their invasion of both RLE cell monolayer and Matrigel did not differ from that of the non-treated (Figure 3B and C). In contrast, when RLE cells were pretreated with MT for 24 h, the invasion of the RLE monolayer by c-SST-2 cells was markedly inhibited (Figure 3A).

**Figure 5** Influence of malotilate on in vitro adhesion of c-SST-2 cells to the rat lung endothelial (RLE) cell monolayer. (**A**) c-SST-2 (open symbols) or RLE (closed symbols) cells were treated with malotilate for 24 h at concentrations of 0 (○), 10 (△, ▲) or 100 (□, ■) ng $ml^{-1}$ before the adhesion assay. (**B**) Both c-SST-2 and RLE cells were pretreated with malotilate for 24 h at concentrations of 0 (○), 4 (●), 20 (▲) or 100 (■) ng $ml^{-1}$, and then the adhesion assay was performed in the presence of malotilate at the same concentration as the pretreatment. Data are expressed as mean ± s.d. from triplicate assay

### Effects of malotilate on the permeability of the rat lung endothelial cell monolayer

The permeability of the RLE monolayer increased by serum starvation when RLE cells seeded on the gelatin-coated membrane of Transwell reached confluence. Pretreatment of the RLE cell monolayer with more than 20 ng $ml^{-1}$ of MT for 24 h led to inhibition of the increase in the permeability by serum starvation to the permeability level of the non-serum-starved RLE monolayer (Figure 4A). The increase in the permeability of the RLE cell monolayer by co-culturing with c-SST-2 cells was also inhibited by pretreatment with MT (Figure 4B).

### Effects of malotilate on in vitro growth, gelatinase production and adhesion to the rat lung endothelial cell monolayer by c-SST-2 and RLE cells

In vitro growth of c-SST-2 or RLE cells was not affected by pretreatment with MT for 24 h (data not shown). The adhesion of c-SST-2 cells to the RLE monolayer was not affected by the pretreatment of c-SST-2 cells or RLE cells with MT (Figure 5A). Furthermore, adhesion of c-SST-2 cells pretreated with malotilate to the RLE cell monolayer also pretreated with malotilate was examined in the presence of the same concentration of molotilate as the pretreatment. As shown in Figure 5B, the adhesion rate of c-SST-2 cells to the RLE monolayer was almost the same even if Malotilate was present during the adhesion assay. By zymographic analysis, 92-kDa, 67-kDa and 64-kDa enzymatic bands were detected in medium conditioned with c-SST-2 cells, and the production of these gelatinases was not changed by MT treatment (Figure 6). No gelatinolytic activity was detected in medium conditioned with RLE cells treated with or without MT.

### DISCUSSION

We showed here that malotilate (MT), which has been used clinically for liver disease, in stimulating liver functions, demonstrated inhibitory action against pulmonary metastasis but not local

**Figure 6** Gelatinase production of c-SST-2 or the rat lung endothelial cells treated with malotilate. Gelatinase production was detected by zymography using gelatin-embedded SDS-PAGE as described in Materials and methods

© Cancer Research Campaign 1998

growth of c-SST-2 rat mammary carcinoma cells. The treatment of c-SST-2 cells with MT did not modify their metastasis-associated properties, such as adhesion and invasion into the RLE endothelial cell monolayer or basement membrane, gelatinase production and migration activity. On the other hand, the in vitro invasion of the RLE monolayer by c-SST-2 cells was inhibited by treatment of RLE but not c-SST-2 cells with MT. Therefore, it seems that MT acts on endothelial cells but not on tumour cells to inhibit metastasis. The inhibitory effect of MT on the in vitro invasion of the RLE monolayer by c-SST-2 cells may be partly because MT prevents the retraction of the RLE monolayer, as MT treatment suppresses the increase in permeability of the RLE monolayer by serum starvation or co-culture with c-SST-2 cells. These findings suggest that MT acts on endothelial cells to intensify the cell adhesion among endothelial cells, which prevents c-SST-2 cells from penetrating through the endothelium.

The inhibitory effect on the experimental metastasis was less than that on spontaneous metastasis. The difference in the effect between experimental metastasis and spontaneous metastasis systems may be caused by the following: (1) in i.v. inoculation of tumour cells, a large number of tumour cells may be circulated at a time, which provides more chances to extravasate endothelium; (2) in the spontaneous metastasis system, tumour cells need to twice penetrate the endothelium that blocks their way, whereas in the experimental metastasis system they need it only once. Thus, inhibitory effects of MT on metastasis may differ depending on inoculation routes of tumour cells.

The mechanism by which MT intensifies cell–cell adhesion among endothelial cells is not clear. It is reported that MT stimulates RNA and protein syntheses and glucose metabolism in the liver and the cultured hepatocyte (Imaizumi, 1982*a* and *b*; Wakasugi and Tomikawa, 1987). It is also known that MT modulates the functions of various types of cells other than hepatocytes (Ryle and Dumont, 1987; Zijlstra et al, 1989; Sunada et al, 1993). In fact, we have observed electron microscopically that MT promoted the development of cell–cell adhesion apparates, such as gap junctions and desmosomes (unpublished data). Further, we have data indicating that the expression of connexin 43 proteins, which are components of gap junctions, is enhanced in RLE cells by MT treatment (unpublished data). Sunada et al (1993) also reported that MT preserved the cobblestone-shaped cell monolayer of bovine endothelial cells for a long-term culture and promoted the formation of adherence junctions and gap junctions between endothelial cells. Therefore, we speculate that MT may stimulate endothelial cells to synthesize proteins, including the molecules involved in homophilic cell–cell adhesion.

Regarding the inhibitory effect of MT on increased permeability of the RLE monolayer caused by co-culture with tumour cells, we also need to consider another possibility besides the intensifying effect of MT on cell adhesion among endothelial cells. Enhanced permeability of the endothelial cell monolayer by co-culture with tumour cells may be due to endothelial cell retraction mediated by the interaction of endothelial cells with the tumour cells. Endothelial cell retraction is known to be induced by soluble factors or direct adhesion of other types of cells from which intracellular signals are transduced to induce morphological changes of cells and to reduce adhesiveness among endothelial cells (Tang et al, 1993*a* and *b*). Therefore, the possibility remains that MT may block the signal transduction involved in endothelial cell retraction, besides intensification of adhesiveness among endothelial cells.

There are some reports that the blockage of the interaction between tumour cells and endothelium in target organs leads to the inhibition of metastasis in animal models. For example, anti-adhesive pepetides based on the amino acid sequence RGDS or YIGSR are able to block the adhesion of tumour cells to subendothelial basement membrane components, resulting in anti-metastatic effects (Humphries et al, 1986; Iwamoto et al, 1987; Saiki et al, 1989). Antibodies to integrins are also known to have similar metastasis-inhibitory effects (Fujita et al, 1992; Kawaguchi et al, 1992). One of the mechanisms for the anti-metastatic action of protease inhibitors, such as tissue inhibitor of metalloproteinase (TIMP) and plasminogen activator inhibitor (PAI), is thought to prevent intra- or extravasation of tumour cells by inhibition of the degradation of the basement membrane (Schultz et al, 1988; Cajot et al, 1990; Albini et al, 1991; Khokha et al, 1992). Anti-angiogenic agents may also be recognized as a blocker of the tumour cell–endothelium interaction to suppress the growth of metastatic foci (Ingber et al, 1990; O'Reilly et al, 1994). Compared with these anti-metastatic agents, MT is unique for its mechanism of action as it enhances the defensive ability of endothelial cells against the penetration by metastatic tumour cells but does not modify the offensive properties of tumour cells per se to establish metastases. As MT did not have any toxicity to SHR rats at the doses used here, it may be potentially useful in the prevention of cancer metastasis, probably being more effective in anti-metastasis when in combination with other therapeutic drugs. However, further study is needed to examine in detail the mechanisms of the intensification of adhesion among endothelial cells and to examine the influence on the other host cells, such as the immune cells.

### ABBREVIATIONS

MT, malotilate; ENDO medium, Dulbecco's modified MEM (DME) supplemented with 10% FBS; RLE, rat lung endothelial cell

### ACKNOWLEDGEMENTS

We thank Ms M Yanome for assistance in preparing the manuscript.

### REFERENCES

Albini A, Iwamoto H, Kleinman HK, Martin GR, Aaronson SA, Kozlowski JM and McEwan RN (1987) A rapid in vitro assay for quantitating the invasive potential of tumour cells. *Cancer Res* **47**: 3239–3245

Albini A, Melchiori A, Santi L, Liotta LA, Brown PD and Stetler-Stevenson WG (1991) Tumor cell invasion inhibited by TIMP-2. *J Natl Cancer Inst* **83**: 775–779

Alvarez OA, Carmichael DF and DeClerck YA (1990) Inhibition of collagenolytic activity and metastasis of tumor cells by a recombinant human tissue inhibitor of metalloproteinases. *J Natl Cancer Inst* **82**: 589–595

Cajot JF, Bamat J, Bergonzelli GE, Kruithof EKO, Medcalf RL, Testuz J and Sordat B (1990) Plasminogen-activator inhibitor type 1 is a potent natural inhibitor of extracellular matrix degradation by fibrosarcoma and colon carcinoma cells. *Proc Natl Acad Sci USA* **87**: 6939–6943

Fujita S, Suzuki H, Kinoshita M and Hirohashi S (1992) Inhibition of cell attachment, invasion and metastasis of human carcinoma cells by anti-integrin β1 subunit antibody. *Jpn J Cancer Res* **83**: 1317–1326

Hamada J, Takeichi N and Kobayashi H (1988) Metastatic capacity and intercellular communication between normal cells and metastatic cell clones derived from a rat mammary carcinoma. *Cancer Res* **48**: 5129–5132

Humphries MJ, Olden K and Yamada KM (1986) A synthetic peptide from fibronectin inhibits experimental metastasis of murine melanoma cells. *Science* **233**: 467–470

© Cancer Research Campaign 1998

Imaizumi Y, Katoh M and Sugimoto T (1982*a*) Effect of malotilate (diisopropyl 1,3-dithiol-2-ylidenemalonate) on the synthesis and movement of RNA in rat liver. *Folia Pharmacol Jpn* **79**: 285–291

Imaizumi Y, Katoh M, Sugimoto T and Kasai T (1982*b*) Effect of malotilate (diisopropyl 1,3-dithiol-2-ylidenemalonate) on the protein synthesis of the rat liver. *Jpn J Pharmacol* **32**: 369–375

Ingber D, Fujita T, Kishimoto S, Sudo K, Kanamaru T, Brem H and Folkman J (1990) Synthetic analogues of fumagillin that inhibit angiogenesis and suppress tumour growth. *Nature* **348**: 555–557

Iwamoto Y, Robey FA, Graf J, Sasaki M, Kleinman HK, Yamada Y and Martin GR (1987) YIGSR, a synthetic laminin pentapeptide, inhibits experimental metastasis formation. *Science* **238**: 1132–1134

Izumi Y, Taniuchi Y, Tsuji T, Smith CW, Nakamori S, Fidler IJ and Irimura T (1995) Characterization of human colon carcinoma variant cells selected for sialyl $Le^x$ carbohydrate antigen: liver colonization and adhesion to vascular endothelial cells. *Exp Cell Res* **216**: 215–221

Kato M and Sugimoto T (1982) Effect of malotilate on chronic liver injury induced by carbon tetrachloride in rats. *Folia Pharmacol Jpn* **80**: 83–91

Kawaguchi S, Kikuchi K, Ishii S, Takada Y, Kobayashi S and Uede T (1992) VLA-4 molecules on tumor cells initiate an adhesive interaction with VCAM-1 molecules on endothelial cell surface. *Jpn J Cancer Res* **83**: 1304–1316

Khokha R (1994) Suppression of the tumorigenic and metastatic abilities of murine B16-F10 melanoma cells in vivo by the overexpression of the tissue inhibitor of the metalloproteinase-1. *J Natl Cancer Inst* **86**: 299–304

Khokha R, Zimmer MJ, Graham CH, Lala PK and Waterhouse P (1992) Suppression of invasion by inducible expression of tissue inhibitor of metalloproteinase-1 (TIMP-1) in B16-F10 melanoma cells. *J Natl Cancer Inst* **84**: 1017–1022

Kobayashi H, Gotoh J, Kanayama N, Hirashima Y, Terao T and Sugino D (1995) Inhibition of tumor cell invasion through Matrigel by a peptide derived from the domain II region in urinary trypsin inhibition. *Cancer Res* **55**: 1847–1852

Laemmli UK (1970) Cleavage of structural proteins during the assembly of the head of bacteriophage T4. *Nature* **227**: 680–685

Liotta LA (1986) Tumor invasion and metastasis – role of the extracellular matrix. *Cancer Res* **46**: 1–7

Liotta LA (1988) Gene products which play a role in cancer invasion and metastasis. *Breast Cancer Res Treat* **11**: 113–124

Mueller BM, Yu YB and Laug WE (1995) Overexpression of plasminogen activator inhibitor 2 in human melanoma cells inhibits spontaneous metastasis in *scid/scid* mice. *Proc Natl Acad Sci USA* **92**: 205–209

Nakajima M, Lotan D, Baig MM, Carralero RM, Wood WR, Hendrix MJC and Lotan R (1989) Inhibition by retinoic acid of type IV collagenolysis and invasion through reconstituted basement membrane by metastatic rat mammary adenocarcinoma cells. *Cancer Res* **49**: 1698–1706

Nicolson GL (1982) Organ colonization and the cell-surface properties of malignant cells. *Biochim Biophys Acta* **695**: 113–176

Nicolson GL (1988) Cancer metastasis: tumor cell and host organ properties important in metastasis to specific secondary sites. *Biochem Biophys Acta* **948**: 175–224

Nicolson GL (1989) Metastatic tumor cell interactions with endothelium, basement membrane and tissue. *Curr Opin Cell Biol* **1**: 1009–1019

Nokata M, Katoh M, Tanaka T and Sugimoto T (1985) Protective effect of malotilate (diisopropyl-1,3-dithiol-2-ylidenemalonate) on carbon tetrachloride-induced liver injury in mice and rats. *J Toxicol Sci* **10**: 279–288

Ohigashi H, Shinkai K, Mukai M, Ishikawa O, Imaoka S, Iwanaga T and Akedo H (1989) In vitro invasion of endothelial cell monolayer by rat ascites hepatoma cells. *Jpn J Cancer Res* **80**: 818–821

O'Reilly MS, Holmgren L, Shing Y, Chen C, Rosenthal RA, Moses M, Lane WS, Cao Y, Sage EH and Folkman J (1994) Angiostatin: a novel angiogenesis inhibitor that mediates the suppression of metastases by a Lewis lung carcinoma. *Cell* **79**: 315–328

Pauli BU, Augustin-Voss HG, El-Sabban ME, Johnson RC and Hammer DA (1990) Organ-preference of metastasis. The role of endothelial cell adhesion molecules. *Cancer Metastasis Rev* **9**: 175–189

Poste G and Fidler IJ (1980) The pathogenesis of cancer metastasis. *Nature* **283**: 139–146

Ryle PR and Dumont JM (1987) Malotilate: the new hope for a clinically effective agent for the treatment of liver disease. *Alcohol Alcoholism* **22**: 121–141

Saiki I, Iida J, Murata J, Ogawa R, Nishi N, Sugimura K, Tokura S and Azuma I (1989) Inhibition of the metastasis of murine malignant melanoma by synthetic polymeric peptides containing core sequence of cell-adhesive molecules. *Cancer Res* **49**: 3815–3822

Schultz RM, Silberman S, Persky B, Bajkowski AS and Carmichael DF (1988) Inhibition by human recombinant tissue inhibitor of metalloproteinases of human amnion invasion and lung colonization by murine B16-F10 melanoma cells. *Cancer Res* **48**: 5539–5545

Sunada H, Masuda M and Fujiwara K (1993) Preservation of differentiated phenotypes in cultured aortic endothelial cells by malotilate and phosphoascorbic acid. *Eur J Cell Biol* **60**: 48–56

Tang DG, Diglio CA and Honn KV (1993*a*) 12(S)-HETE-induced microvascular endothelial cell retraction results from PKC-dependent rearrangement of cytoskeletal elements and $\alpha v\beta 3$ integrins. *Prostaglandins* **45**: 249–268

Tang DG, Timar J, Grossi IM, Renaud C, Kimler VA, Diglio CA, Taylor JD and Honn KV (1993*b*) The lipoxygenase metabolite, 12(S)-HETE, induces a protein kinase C-dependent cytoskeletal rearrangement and retraction of microvascular endothelial cells. *Exp Cell Res* **207**: 361–375

Wakasugi J and Tomikawa M (1987) Stimulatory effect of malotilate on protein synthesis in hepatocytes. In *New Trends in Peptic Ulcer and Chronic Hepatitis. Part II. Chronic Hepatitis*, The Japanese Society of Gastroenterology, (eds). pp. 342–346. Excerpta Medica: Tokyo

Zijlstra FJ, Wilson JHP, Vermeer MA, Ouwendijk RJT and Vincent JE (1989) Differential effects of malotilate on 5-,12- and 15-lipoxygenase in human ascites cells. *Eur J Pharmacol* **159**: 291–295

© Cancer Research Campaign 1998